# An Occlusion-Aware Framework for Real-Time 3D Pose Tracking

**DOI:** 10.3390/s18082734

**Published:** 2018-08-20

**Authors:** Mingliang Fu, Yuquan Leng, Haitao Luo, Weijia Zhou

**Affiliations:** 1 State Key Laboratory of Robotics, Shenyang Institute of Automation, Chinese Academy of Sciences, Shenyang 110016, China; luohaitao@sia.cn (H.L.); zwj@sia.cn (W.Z.); 2 University of Chinese Academy of Sciences, Beijing 100049, China; 3 Department of Mechanical and Energy Engineering, Southern University of Science and Technology, Shenzhen 518055, China; lengyq@sustc.edu.cn

**Keywords:** pose tracking, occlusion handling, online rendering, motion compensation

## Abstract

Random forest-based methods for 3D temporal tracking over an image sequence have gained increasing prominence in recent years. They do not require object’s texture and only use the raw depth images and previous pose as input, which makes them especially suitable for textureless objects. These methods learn a built-in occlusion handling from predetermined occlusion patterns, which are not always able to model the real case. Besides, the input of random forest is mixed with more and more outliers as the occlusion deepens. In this paper, we propose an occlusion-aware framework capable of real-time and robust 3D pose tracking from RGB-D images. To this end, the proposed framework is anchored in the random forest-based learning strategy, referred to as RFtracker. We aim to enhance its performance from two aspects: integrated local refinement of random forest on one side, and online rendering based occlusion handling on the other. In order to eliminate the inconsistency between learning and prediction of RFtracker, a local refinement step is embedded to guide random forest towards the optimal regression. Furthermore, we present an online rendering-based occlusion handling to improve the robustness against dynamic occlusion. Meanwhile, a lightweight convolutional neural network-based motion-compensated (CMC) module is designed to cope with fast motion and inevitable physical delay caused by imaging frequency and data transmission. Finally, experiments show that our proposed framework can cope better with heavily-occluded scenes than RFtracker and preserve the real-time performance.

## 1. Introduction

3D pose tracking of a rigid object in an image sequence is one of the most core research fields in computer vision. Different from the object tracking in the image plane [1,2,3], pose tracking in 3D means retrieving the 3D translation and rotation of an object in the camera coordinate system. It serves as a cornerstone for numerous computer vision applications, such as augmented reality [4], robotic interaction [5] and medical navigation [6]. Until the advent of RGB-D sensors, early pose tracking mostly adopted a template matching-based strategy [7] or correspondences between natural landmarks [8]. Making it easy to capture 3D information of the scene, consumer RGB-D cameras break fresh ground for the more rapid development of pose tracking [9,10,11,12,13]. Marked improvements have been achieved in almost every aspect, from dense feature fields [14], 3D signed distance functions [15] and pixel-wise optimization [12], to random forests-based learning [16,17] (named as RFtracker below), deep feature [18] and hybrid energy optimization [19].

Related strategies can be roughly divided into three categories according to the data type of input: (1) RGB image-based methods [13,20]; (2) depth image-based methods [16,17]; (3) RGB-D-based methods [21,22,23]. In the case of 3D pose tracking from RGB images, prior shape knowledge is often the necessary input. A probabilistic model connecting statistical appearance models of background and foreground and 3D pose parameters was built in [20]. Pose tracking was derived by optimizing global appearance-based energy. Based on this probabilistic model, an energy function using multiple local appearance models [13] was proposed to capture the spatial variation in statistical properties. In addition to probabilistic model-based approaches, building the local correspondence is an alternate choice for only the color cue. For the case where depth images are available, learning-based methods with random forest [16,17] comprise a state of the art framework. Tan et al. [16,17] originally proposed to build the mapping between displacement vectors and temporal parameters. With both color and depth cues, the options become diverse. Hinterstoisser et al. [23] presented a robust template-based approach with the color gradient and surface normal. Choi et al. [22] fed multiple geometric features to a particle filter. Beyond these, the dense 3D object coordinate [21] has also been proven to be an effective feature for correlating pose parameters.

For most applications in this community, real-time performance is the necessary attribute for trackers. Before proceeding, we first clarify the two mechanisms: tracking-by-detection [18,21] and temporal tracking [16,17,20,22]. The former assumes that sequence frames are independent and performs pose detection for each frame. In contrast, the input of temporal tracking consists of the live frame and previous pose parameters. Existing temporal trackers achieve real-time tracking gracefully by virtue of novel approximations [19] or theoretical efficiency [16,17]. With the help of GPUs’ powerful geometric computing ability, methods suitable for parallel computing such as the partial filter [22] reach the real-time level. Occlusion handling is another essential skill for pose tracking. The built-in occlusion handling [16,17] and sparse approximations [19] enable these methods to cope with occlusion under low levels. To improve the robustness against occlusion, Tan et al. [17] proposed a passive strategy that the tracker learns occlusion handling from predetermined occlusion patterns, which represent the distribution of random selected tracking points. Obviously, this random strategy does not always model the real scene well. Besides, increasing trees in the forest are disturbed inevitably as the occlusion deepens. Meanwhile, RFtracker directly uses the selected individual trees, which are trained separately to predict the forest’s output. Suboptimal learning loss [24] caused by the heuristic learning rule of random forest is ignored in RFtracker [17].

In this paper, we focus on an occlusion-aware and real-time framework for 3D temporal tracking. To this end, we propose a framework built upon RFtracker and introduce two necessary improvements to enhance tracking accuracy and robustness to occlusion. On the one hand, an active occlusion handling strategy built on online rendering and depth comparison is proposed. Thus, random forest in our framework will receive filtered data instead of raw displacement vectors. Depth comparison in occlusion handling is built on the assumption that the update transformation between consecutive frames is not large. However, fast motion and inevitable physical delay caused by the imaging process and data transmission make this assumption less stable. For this purpose, a lightweight convolutional neural network (CNN)-based motion compensation module is designed to bridge the gap between consecutive frames. A local refinement is presented to improve the forest’s regression on the other. Note that random forest with local refinement is superficially similar to multi-forest [16]. The core difference is that we mix complementary information between individual trees into the final prediction.

To summarize, the main contributions of this paper are highlighted as follows:

1. To guide random forest towards optimal regression, a new loss function considering complementary information between individual trees is defined.
2. An online rendering-based occlusion handling method is proposed to improve the robustness against occlusion.
3. A lightweight convolutional neural network-based motion compensation module is presented to cope with jitter caused by fast motion and physical delay.

The rest of this paper is structured as follows: In Section 2, the details of the proposed framework are presented. Next, in Section 3, we present the implementation details and our results compared with RFtracker. Conclusions and future work are given in Section 4.

## 2. Method

**Figure 1:**
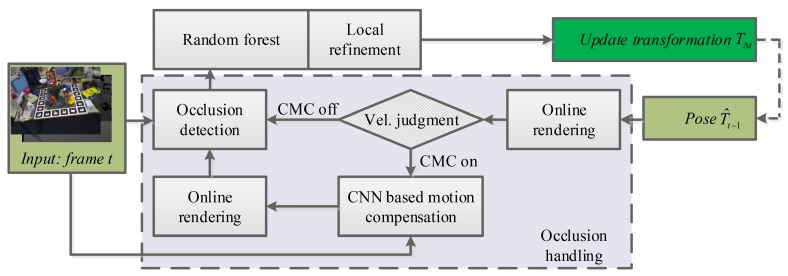
Pipeline of the proposed occlusion-aware framework. CMC, convolutional neural network-based motion-compensated.

The proposed occlusion-aware framework is detailed in this section. As shown in Figure 1, the input of the proposed framework consists of previous pose parameters and the live RGB-D image pair $I_{t}$ and $D_{t}$. After dataflow travels over the forest, the final output is a 6-DoF update transformation between consecutive frames. To close the loop, an online rendering-based occlusion handling module is employed to connect the update transformation and input frame. As a key component of occlusion handling, the convolutional neural network-based motion-compensated (CMC) module takes the synthesized template and live frame as input and outputs the motion compensated transformation. Once having received the update transformation, the rendering engine prepares an occlusion-free template for occlusion detection. Thus, the interference from the occluded area can be alleviated, and random forest can focus more on building the mapping between displacement vectors and pose parameters. The main reason to introduce the CMC module is to compensate the low overlap rate between sequential inputs. That is, the CMC module is activated only when the frame-to-frame transformation is larger than a preset threshold. Therefore, a switch-based velocity judgment is integrated into the framework to control the turning on and off of the CMC module.

### 2.1. Random Forest-Based 3D Temporal Tracker

Random forest [25] is an ensemble learning method with many ideal attributes. In consideration of its powerful non-linearity fitting and high efficiency, Tan et al. [17] proposed a 3D temporal tracker anchored in random forest. Similar to model-based tracking [26], a view sphere centered on a 3D model is constructed. To generate the training dataset, displacement vectors of randomly selected tracking points and the corresponding update transformation are accumulated for each viewpoint. In the forest, one tree is responsible for a unique viewpoint. After test data travel over the forest, final predictions are calculated by averaging adjacent trees. Note that predictions of the forest are the object’s update transformation between consecutive frames. During the training or testing stage, each tree is responsible for one parameter, and each viewpoint has an individual unit consisting of 6 trees. Three of them are for 3D translation, and the rest for 3D rotation.

### 2.2. Local Refinement of Random Forest

**Figure 2:**
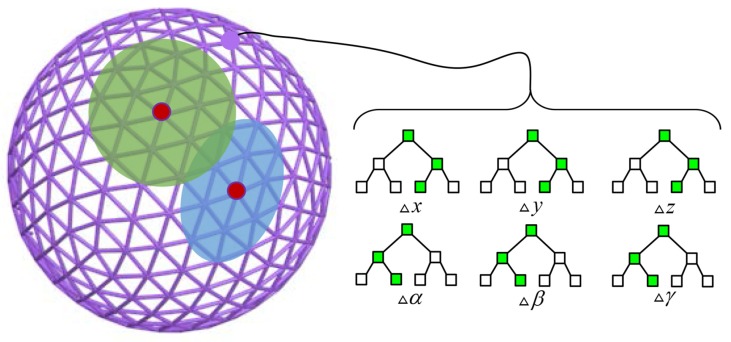
View sphere in RFtracker. Each vertex on the view sphere represents a camera viewpoint. Trees inside the blue region and the green region are selected for the final prediction in the testing stage, respectively. The viewpoint corresponding to the red point located at the region’s center is determined by the previous pose.

As shown in Figure 2, the view sphere is divided into an icosahedron with $N_{v}$ vertices, and each vertex represents a camera view. An exclusive sub-dataset consisting of displacement vectors and frame-to-frame transformations is assigned for each camera view. Given the previous transformation $T_{t-1}$ and depth frame $D_{v}$ of the *v*-th camera view, the corresponding displacement vector can be computed by Equation (1).

(1) $$\varepsilon \;{\left(T_{t-1},D_{v}\right)}_{t}=n_{v}\cdot \left(T_{t-1}^{-1}\pi \left(x\right)-X\right)$$

where $n_{v}$ is a unit vector pointing to the origin of the camera coordinate system, π is the re-projection function and *x* and *X* represent a tracking point’s projection in the image coordinate system and the location of a tracking point in the object coordinate system, respectively. Note that the unit vector $n_{t-1}$ is computed with the previous object transformation $T_{t-1}$. As described above, each tree in the forest is trained independently with its own sub-dataset. The forest’s prediction is computed by averaging selected trees. Complementary information between individual trees is thus not employed during the prediction. Loss functions defined on the training and testing are obviously inconsistent. To remove this inconsistency, an ideal loss function that takes into account the complementary information among individual trees needs to be redefined.

Regardless of extreme fast motion, update transformations across sequence images change smoothly along the time line. In the view sphere, object transformations belonging to adjacent viewpoints are approximate. In consideration of the above two points, trees located at adjacent viewpoints are selected to predict jointly update transformation, and the rest are put aside temporarily. For the RFtracker, these selected trees are treated as separate individuals in the training, and thus, forests are not guided to toward the optimal direction. To address this issue, we create a tuple consisting of $N_{nt}$ neighborhood trees for each viewpoint. Inside each tuple, a single camera view still uses 6 trees to store training parameters. This expanded forest contains $N_{v}$ tuples, a total of $6N_{nt}\cdot N_{v}$ trees. Trees inside each tuple are jointly trained under a local loss function and predict the update transformation collaboratively, which is the key difference between this extended forest and the RFtracker. It should be noted that tuples with adjacent viewpoints are refined separately to avoid mutual interference from the overlapping neighborhood.

**Figure 3:**
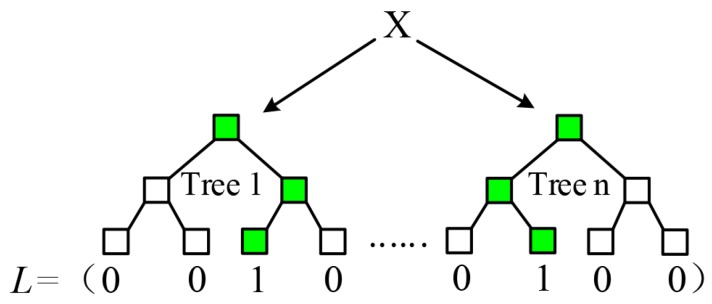
The process to determine elements of an indicator vector. The blue leaf or non-leaf nodes in forest represent data stream. Input data travel over the trees and are finally stored in the blue leaf nodes. After that, an indicator vector with the same dimension as the number of leaves is determined. Each element in the indicator vector depends on whether the corresponding leaf node contains the input data or not.

Essentially, random forest builds a non-linear mapping between sample data and corresponding labels. Ren et al. [24] proposed to utilize an indicator vector (see Figure 3) $\phi \left(x\right)$ to specify this mapping $y=w\phi \left(x\right)$, where *w* is the leaf matrix packing all leaf nodes. Analogously, the mapping associating displacement vector ε with temporal parameters $T_{\Delta t}$ can be formulated as Equation (2).

(2) $$T_{\Delta t}=\mathrm{Tree}\left(\varepsilon_{t},\varphi \right)=\omega \psi \left(\varepsilon_{t},\varphi \right)$$

where $\psi \left(\cdot \right)$ denotes an improved indicator vector with an adjustable ratio φ used for selecting leaf nodes with a lower standard deviation and ω is the leaf matrix consisting of parameter means stored in leaf nodes. Note that the corresponding indicator of leaf nodes with a larger standard deviation will be set to 0.

#### 2.2.1. Learning with Indicator Vector

For the *v*-th viewpoint, the corresponding tuple consists of neighborhood trees determined by an angle threshold between the unit vector $N_{t-1}$ and adjacent camera views. During temporal tracking, the estimated transformation at timestamp *t* satisfies ${\hat{T}}_{t}=T_{\Delta t}{\hat{T}}_{t-1}$ where $T_{\Delta t}$ denotes the regressor of random forest from timestamp *t*. Therefore, a series of regressors from random forest are predicted to drive temporal tracking. After the extension of the forest, its corresponding training process is also slightly different. Once input data reach the leaf nodes of the forest, indicator vectors $\psi_{i}$, $i=1,2,\ldots ,N_{nt}$ from different trees are determined simultaneously. Then, the *v*-th tuple is determined by the unit vector $N_{t-1}$; all the indicator vectors and leaf matrices inside this tuple are packed to form a high-dimensional binary feature and leaf matrix, as shown in Equation (3).

(3) $$\begin{array}{c} L\left(\varepsilon ,\varphi \right)=\left[\psi_{1},\psi_{2},\cdot \cdot \cdot ,\psi_{N_{nt}}\right],\\ W=\left[\omega_{1},\omega_{2},\cdot \cdot \cdot ,\omega_{N_{nt}}\right],\\ \omega_{i}=\left[\left(m_{1},\sigma_{1}\right),\left(m_{2},\sigma_{2}\right),\cdots ,\left(m_{N_{i}},\sigma_{N_{i}}\right)\right] \end{array}$$

where *W* represents the leaf matrix consisting of the mean $m_{i}$ and standard deviation $\sigma_{i}$ of transformation parameters stored in leaf nodes and $N_{i}$ represents the total number of leaf nodes in *i*-th tree.

#### 2.2.2. Learning with the Leaf Matrix

For RFtracker, the objective function of random forest in learning can be written as Equation (4).

(4) $$\begin{array}{c} \min_{\omega_{i}}\frac{1}{N_{vp}}\sum_{i=1}^{N_{vp}}sum\left(\theta_{i}^{sd}\right)\\ s.t.\;\theta_{i}^{sd}=\omega_{i}\psi_{i}\left(\varepsilon \right),\;\forall i\in \left[1,N_{vp}\right] \end{array}$$

where $N_{vp}$ denotes the number of viewpoints across the view sphere and $sum\left(\cdot \right)$ is the error function defined on the tree’s prediction $\theta_{i}^{sd}$, which is a vector collecting the standard deviation stored in leaf nodes.

Individual trees are obviously trained separately without sharing the complementary information with adjacent trees. The loss function of RFtracker in learning is a simple average over individual trees’ loss, which is irrelevant to the final predictions of the forest. Next, we present the reformulated version with consideration of the complementary information inside the tuple. After the indicator vector and the leaf matrix are determined, these data are not used directly to calculate forest’s regressors. The leaf matrix is discarded instead of being used to compute the final prediction. Formally, the leaf matrix *W* of the *v*-th tuple is re-learned by minimizing the following loss:

(5) $$\begin{array}{c} \min_{W}\sum_{v=1}^{N_{vt}}\sum_{i=1}^{N_{v}}{∥\theta_{i}^{gt}-\theta_{i}^{est}∥}_{2}^{2}+\lambda {∥W∥}_{2}^{2}\\ s.t.\;\theta_{i}^{est}=WL\left(\varepsilon_{i},\varphi \right) \end{array}$$

where λ controls the tradeoff between training loss and the regularization term, $N_{v}$ denotes the total number of training samples stored in the *v*-th tree, $\theta_{i}^{gt}$ and $\theta_{i}^{est}$ denote the ground truth labels and the corresponding estimated regressors, respectively. It should be noted that the synthetic depth image and transformation set that correspond to the *v*-th tree are applied to all the $N_{vt}$ trees in this tuple.

However, it has been found that overfitting occurs from time to time when using the above local refinement. The prediction model described by Equation (5) is dependent on training data unduly and has a high error rate on the unseen data. That is, some of the discarded predictions have lower error than refined predictions, and the fitting capacity of individual trees is weakened. Alternatively, alternating regression forests (ARFs) [27] interrelate the complementary information between individual trees during a stage-wise training. Several weak learners $h_{d}\left(x\right)$ are combined to generate a stronger learner F(x) in turn. Each depth *d* corresponds to a single stage. More specifically, a new weak learner such as a random tree is added to the previous prediction to generate a stronger learner $F_{d}\left(x\right)=F_{d-1}\left(x\right)+h_{d}\left(x\right)$. ARFs utilize a more elaborate method, which applies gradient descent to the function space to correlate the negative gradient of the loss function and the current output. For the training sample $\left(\varepsilon ,\theta_{gt}\right)$, the corresponding pseudo target [28] can be calculated by Equation (6).

(6) $$-g_{d}\left(\varepsilon \right)=-{\left[\frac{\partial Loss\left(\theta_{gt},F\left(\varepsilon \right)\right)}{\partial F\left(\varepsilon \right)}\right]}_{F\left(\varepsilon \right)=F_{d-1}\left(\varepsilon \right)}$$

In a real case, the pseudo targets act as the error of the existing prediction model and can be replaced with the difference between the ground truth and previous estimate $F_{d-1}\left(x\right)$. Given the previous prediction $F_{d-1}\left(x\right)$ at depth d−1, ARFs can influence the training in the next stage via updating the global loss based on Equation (6). By comparing the above two ways of mining complementary information, it can be found that ARFs reserve predictions of individual trees, while interrelating the complementary information and relearning-based strategy [24] does not change the trees’ structure. To inherit advantages of both methods simultaneously, a modified prediction model is pointedly introduced through numerical optimization in the function space, as denoted by Equation (7).

(7) $$\begin{array}{c} \min_{W}\sum_{v=1}^{N_{vt}}\sum_{i=1}^{N_{v}}{∥-g_{d}\left(\varepsilon \right)-\theta_{i}^{est}∥}_{2}^{2}+\lambda {∥W∥}_{2}^{2}\\ s.t.\;\theta_{i}^{est}=WL\left(\varepsilon_{i},\varphi \right) \end{array}$$

where the unconstrained negative gradient $-g_{d}\left(\varepsilon \right)$ is approximated by the difference between the ground truth $\theta_{gt}$ and forest regressor $F_{d-1}\left(\varepsilon ,\varphi \right)=\frac{1}{N_{t}}\sum_{i=1}^{N_{t}}Tree_{i}\left(\varepsilon ,\varphi \right)$.

Leaf nodes of the original forest correspond to the previous prediction $F_{d-1}\left(x\right)$ trained up to the stage d−1, and the refinement strategy described by Equation (7) corresponds to the training depth *d*. Note that the final prediction at depth *d* is the pseudo targets instead of the ground truth labels. We would like to stress that the leaf matrix corresponding to this modified prediction model will be added to the forest’s prediction at depth d−1 to obtain the final result. That is, a local refinement is performed to compensate the error of the existing prediction model, and we refine the prediction model of RFtracker instead of the relearning-based strategy. The minimization described by Equation (7) can be handled well by the liblinear library [29]. It should be noted that although the local refinement utilizes more than 70,000 trees in the learning stage, only trees in a single tuple are active simultaneously in the prediction. Thus, the time overhead to complete a prediction loop is still close to the original method. So far, extra computation comes mainly from the leaf optimization.

### 2.3. CNN-Based Motion Compensation

As mentioned earlier, the CMC module is designed to handle occlusion. Someone may argue that an occlusion-free template can be rendered directly by using the regression-derived pose. Although RFtracker has shown extreme low computational cost and good tracking accuracy, it still suffers from physical restrictions in real applications. Specifically, pose stream is inevitably mixed with physical delay such as the imaging process, exposure time and data transmission. In order to overcome the above problems, the CMC module is presented to bridge the gap.

#### 2.3.1. Network Architecture

**Figure 4:**
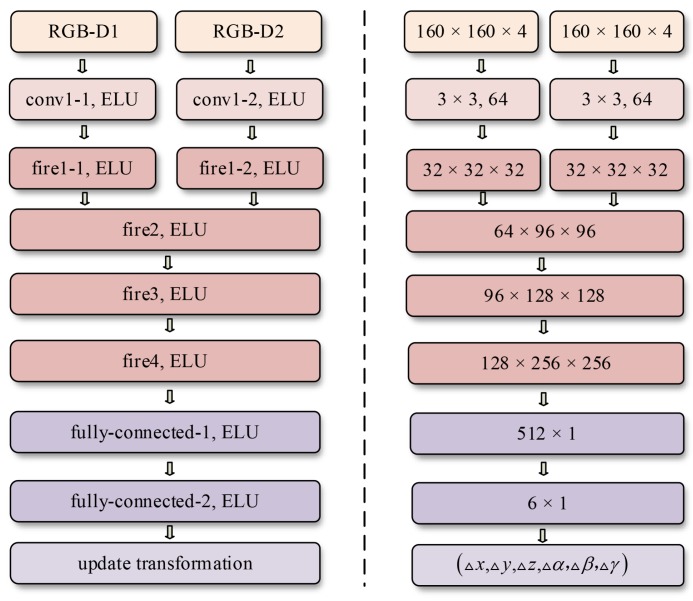
Architecture (left) and component size (right) of the CMC module. The early layer consists of two collateral convolutional layers with 64 3×3 filters. Fire modules (in crimson) [31] in the network architecture are employed to replace conventional convolutional layers. Three hyper-parameters of the fire module represent the number of filters in the squeeze layer, the number of 1×1 filters and the number of 3×3 filters in the expand layer, respectively. For the input convolutional layers conv1-1 and conv1-2, the stride is set to 2.

To achieve rapid inference, we utilize a simple neural network structure similar to a Siamese network [30]. As shown in Figure 4, the network takes two resized RGB-D frames as the input. Before merging two collateral inputs, they will pass through one convolutional layer and fire module. To maintain competitive accuracy with few parameters, SqueezeNet [31] uses numerous 1×1 filters to replace 3×3 filters and decrease the number of input channels to 3×3 filters. Meanwhile, downsampling is performed at late layers to ensure that the convolutional layers can have large activation maps. Based on the above design strategies, the typical fire module is comprised of a squeeze convolution layer and an expand layer. A squeeze layer usually has only 1×1 filters, of which the output is passed to the expand layer consisting of 1×1 and 3×3 filters. In view of the impressive performance of model compression, fire modules instead of convolutional layers are utilized to deepen the network. Such an early fusion can avoid excessive loss of related spatial information as the network deepens. After the fusion, the data stream will go through three fire modules and two fully-connected layers embedded at the end. Each convolutional layer is followed by a pooling layer and a nonlinear activation function. With the rapid development of the deep learning community, we have many alternative activation functions such as parametric rectified linear unit (PReLU) [32], rectified linear unit (ReLU) [33,34] and exponential linear unit (ELU) [35]. ELU finally wins the bid because of better performance in terms of convergence speed and accuracy in our tests. The cost function is built by comparing the predicted transformation vector with the corresponding ground truth label. In order to avoid damaging the learning dynamics, early stopping [36] is employed as a form of regularization and the training data are split for training and validation into a ratio of 3:1. That is, the no-improvement-in-*n* strategy is activated if the validation loss has not been improved in the next *n* epochs.

#### 2.3.2. Training Dataset

For RFtracker, the training dataset consists of a series of displacement vectors and corresponding transformation vectors. To extend input pairs of random forest, random transformation vectors sampled over a fixed range are applied to selected tracking points to mimic locations from the previous frame. Displacement vectors of these tracking points are packed to personalize trees in random forest. For the proposed framework, two different training datasets are needed obviously. One is for random forest, and the other is for the CNN in CMC module. Apparently, it seems that we should utilize the combined dataset to optimize leaf nodes. However, upon closer inspection of the training dataset, we find that this simple combined strategy is not applicable. The training dataset is a consistent whole in a standard random forest. However, for the RFtracker, the training dataset is evidently a form of viewpoint-specific data. Besides, the introduction of local refinement does not alter the structure of the pre-trained forest. Given these characteristics, the random forest in our proposed framework can share the same training dataset with RFtracker.

As the input of RFtracker, displacement vectors can represent the spatial relationship of the tracked object between consecutive frames. Instead of learning from discrete data like random forest, we try to build a comprehensive mapping between the image domain and numerical labels with CNN’s powerful representation ability. Inspired by mappings built via random forest, we utilize two synthetic frames instead of the displacement vector and the corresponding update transformation to form a basic unit of the training dataset. Here, a certain number of consecutive image pairs instead of displacement vectors is employed to train the CNN in the CMC module.

For a tracked object with 3D model available, we firstly generate 180,000 RGB-D frames of which the rendering engine randomly samples a transformation with a radius that ranges in $\left[400\;\mathrm{mm},\;1600\;\mathrm{mm}\right]$ and polar angles in $\left[-180^{\circ },\;180^{\circ }\right]$ in the spherical coordinate system. The corresponding rigid transformations over rendered frames are referred to as live poses. After that, the inverse of a random transformation is applied to the live pose for generating a simulative previous frame. This random transformation samples from a translation that ranges in $\left[-20\;\mathrm{mm},\;20\;\mathrm{mm}\right]$ and angles that range in $\left[-15^{\circ },\;15^{\circ }\right]$.

#### 2.3.3. Data Preprocessing

**Figure 5:**
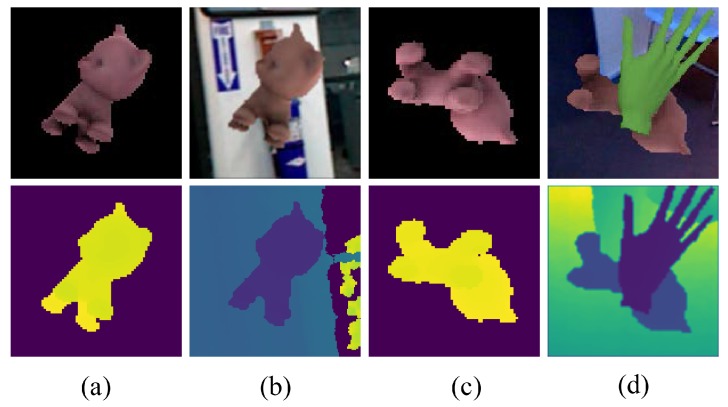
Exemplary training samples used in the CMC module. Color (up) and depth (down) image pairs (**a**) and (**c**) are synthesized by feeding a random pose to the rendering engine. (**b**) and (**d**) are the corresponding image pairs after augmentation operations and stacking relative transformations.

A tracked object may appear anywhere in the image plane during tracking. To avoid biasing the network, we define a region of interest determined by object transformation and resize it to 160×160 in both color and depth frames. Note that the object transformation is replaced with the given previous transformation. For improving the robustness against perturbations, we employ different kinds of data augmentation, such as changing brightness, background, noise and occlusion. After these above steps, both color and depth images are normalized by the statistics of color and depth channels among the whole training dataset. Some training samples are shown in Figure 5.

### 2.4. Online Rendering-Based Occlusion Detection

**Figure 6:**
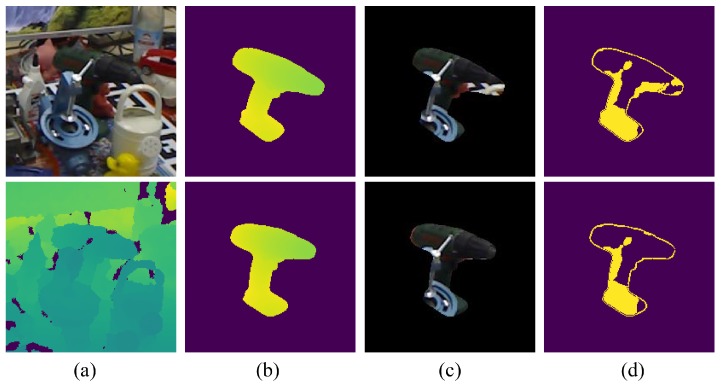
An example of occlusion detection with the depth map. RGB images are provided for better visualization. The test sample from the Occluded LineModdataset [21] is employed to show the flow of occlusion detection. In (**a**), RGB image crop (up) and depth image crop (down) of an occluded scene are displayed. The synthetic depth maps (**b**) and projected mask (**c**) on the RGB image are generated with the compensated pose (up) and ground truth pose (down), respectively. The labeled region corresponds to low-quality area shown in (**d**).

Occlusion detection and handling remain an open problem at all times in tracking [3]. Starting from an intuitive point of view, the occlusion area is nearly identified with an available occlusion-free template. Towards this direction, the compensated transformation parameters are passed to the rendering engine for a background-free and occlusion-free template. Similar to [37], the object region of the non-occluded template is compared to the test frame to determine the occluded pixels. This non-occluded template makes it easy to get out of background clutter and activates many potential methods. A color-based comparison builds on the time-consuming illumination estimation [38,39] and falls into perplexity when the occluder and tracked object have a similar color. Another observation is that occluders always appear between the tracked object and the observer in most cases, and thus, they usually have different distance attributes. Given these details, a depth map instead of a color image is employed to handle dynamic occlusion. A simple and effective occlusion detection method is designed based on the observation. With preparatory work completed, we have the motion-compensated depth map $D_{mc}$ and test frame $D_{new}$ at hand. RFtracker learns a built-in occlusion handling capacity with tracked points randomly selected from the target mask. For the test frame $D_{new}$, non-occluded pixels inside the target mask correspond to the high-quality sampling region. On the contrary, the low-quality region $S_{L}$ refers to non-target appearance such as occluded pixels and background. To determine the low-quality region $S_{L}$, we apply a two-step strategy to the test frame. The corresponding detection can be detailed as follows:

1. Depth comparison-based occlusion and background detection: Although the rendering template with motion-compensated transformation bridges the gap between sequential frames, a direct pixel-wise comparison is still inexact because of motion blur and a noisy depth map. To improve detection efficiency, we utilize the processed images with Gaussian blur instead of raw inputs. In view of the random distribution of tracking points, as many occluded pixels as possible should be labeled. To this end, the dilation operation is embedded in the backend of coarse detection. In our implementation, both the rendering template and test frame are convolved by the 6×6 Gaussian kernel $G^{\sigma }$ with a standard deviation of 5. To reduce time cost, filtering is only performed in the region of interest (ROI) determined by the motion-compensated pose and the object’s diameter. Formally, the occluded region identified by depth images can be expressed as Equation (8).
  (8) $$S_{D}=Mask\left(\left|G^{\sigma }*D_{new}^{ROI}-G^{\sigma }*D_{mc}^{ROI}\right|-\beta \right)⊕E_{D}$$
  where Mask(·) labels pixels with a higher difference than threshold β, ∗ denotes the filtering operation, $E_{D}$ represents a quadrate structuring element with a side of 3 pixels, ⊕ is an image dilation operator for expanding the detected occlusion and $D_{new}^{ROI}$ and $D_{mc}^{ROI}$ represent the ROI in the test frame and motion-compensated frame, respectively.
2. Projected silhouette of the rendering template: As previous literature reported, raw depth maps captured by Kinect meet interferences such as sensor noise [40] or flying pixels [41] at depth discontinuity. The difference between the compensated pose and ground truth pose exacerbates the unreliability. Thus, the silhouette of the non-occluded template is labeled in this stage. As shown in Figure 6, some background pixels appear in the mask area. The projected silhouette of the tracked object is added to the low-quality region to maximize purification of the forest’s input. We can easily label the silhouette pixels $S_{e}$ with an occlusion-free and background-free template. Similar to the coarse detection, the dilation operation is applied to the detected silhouette. The final low-quality region can be determined by combining the silhouette pixels $S_{e}$ with the occluded region $S_{D}$ (see Equation (9)).
  (9) $$S_{L}=S_{D}\cup \left(S_{e}⊕E_{D}\right)$$

From Figure 6c, the projection mask on the RGB image that corresponds to the compensated pose contains more background pixels. By contrast, occlusion in Figure 6d can be detected almost perfectly with the ground truth pose. However, if without the CMC module, the projection mask will contain more low-quality areas dominated by background pixels or occluders, which frequently leads to tracking loss. Note that once the occlusion detection is completed, all indexes of the low-quality region are stored for filtering test data of random forest.

### 2.5. Details of Using Detected Occlusion

Before showing how to exploit the detected occlusion, we firstly review how to learn occlusion handling in RFtracker. RFtracker achieves a built-in occlusion handling capability from sparse tracking. A certain number of sample points is randomly selected from the target region to generate displacement vectors. As the level of occlusion increases, displacement vectors are mixed with an increasing number of outliers. The result is that regression accuracy declines sharply, and even losses of the tracking occur frequently. Therefore, the key to maintain and improve prediction accuracy is to distinguish occluded sample points from test data. In the proposed framework, tracked points stored in activated trees will be filtered before traveling over the forest. More specifically, a displacement vector is set to 0 if the corresponding tracked point falls into the low-quality region after back-projection.

## 3. Experiments

The proposed framework is written in python and runs on a core i7@3.5 GHz (Intel, Santa Clara, CA, USA) and NVIDIA GTX 1080Ti (NVIDIA, Santa Clara, CA, USA). The CMC module is built on PyTorch [42], which is an open source machine learning library with modular components of a deep learning architecture. A synthetic dataset [22] consisting of four motion sequences each containing 1000 frames with the ground truth pose is utilized to evaluate the reimplementation. The LineMod dataset [23] and the occluded version [21] are employed to evaluate the tracking accuracy and robustness against occlusion.

The proposed framework builds on RFtracker, and thus, parameter settings of random forest are reserved. That is, the view sphere is equally divided into 642 camera views, and training data for each tree consist of 2500 samples. The angle threshold for determining neighborhood trees is set to $35^{\circ }$. Beyond these, the optimal parameter λ for the local refinement is set to $10^{-4}$ after a cross-validated grid search. Adam [43] is utilized to optimize the loss of CMC module, with a learning rate of $10^{-5}$ and mini-batches of 64 image pairs.

### 3.1. Lifting of the Overlap Ratio

**Figure 7:**
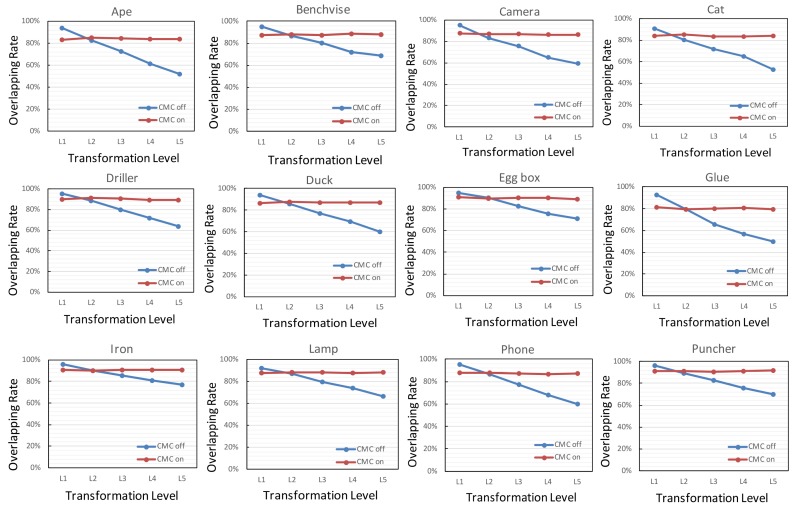
Overlapping ratio between the rendering template and tracked object in a real scene.

In the proposed framework, the CMC module is designed to increase the overlap ratio between the rendering template and tracked object in the test frame. It is obvious that the overlapping ratio is negatively correlated with the update transformation between sequential frames. Given a test frame and rendering template, the overlapping ratio can be determined by Equation (10).

(10) $$overlapping\_ratio=\frac{N_{render}}{N_{scene}}$$

where $N_{scene}$ is the total number of pixels inside the projected mask of a LineMod object in the test frame and $N_{render}$ is the number of synthetic pixels located in the corresponding region determined by the same mask. In order to evaluate the impact of the CMC module on the overlapping ratio, the update transformation is sampled from a Euler angle in the range $\left[-15^{\circ },\;15^{\circ }\right]$ and a translation in the range $\left[-20\;\mathrm{mm},\;20\;\mathrm{mm}\right]$ for each axis. For a quantitative comparison, the update transformation applied to the LineMod sequences is divided into five levels (both translation and rotation are represented in an absolute value): $L1=[0^{\circ }/0\;\mathrm{mm},3^{\circ }/4\;\mathrm{mm}]$, $L2=[3^{\circ }/4\;\mathrm{mm},6^{\circ }/8\;\mathrm{mm}]$, $L3=[6^{\circ }/8\;\mathrm{mm},9^{\circ }/12\;\mathrm{mm}]$, $L4=[9^{\circ }/12\;\mathrm{mm},12^{\circ }/16\;\mathrm{mm}]$ and $L5=[12^{\circ }/16\;\mathrm{mm},15^{\circ }/20\;\mathrm{mm}]$. For each tracked object, average values of the overlapping ratio on a single LineMod sequence are recorded. The corresponding plots of 12 LineMod sequences are shown in Figure 7.

As can be seen in Figure 7, the overlapping ratio of LineMod sequences decreases with the increase of the transformation level when the CMC switch is off. The CMC module increases the overlapping ratio under all four higher levels. Besides, the overlapping rate of LineMod sequences can always be maintained at a consistent level despite the increasing motion level. Note that when the update transformation between sequential frames lies at a lower level, such as the L1 level, the rendering template derived by previous transformation is close to the real projected silhouette in the test frame. For all LineMod objects, the addition of the CMC module will slightly reduce the overlapping ratio at lower levels. Here, the overlapping ratio is a statistical average value, which indicates that nearly half of the test frames have an overlapping ratio below the average. A low overlapping ratio will result in a decrease of the valid input data of random forest, a decline in tracking accuracy or even tracking loss.

At a low level of frame-to-frame motion, the CMC module achieves a very limited compensation and even reduces the overlapping ratio of parts of LineMod objects. Thus, the threshold of translation and rotation of the CMC switch is set to $6^{\circ }$ and 8 mm.

### 3.2. Tracking Accuracy on the RGB-D Object Pose Tracking Dataset

To investigate the efficiency of local refinement and occlusion detection, we specify two different implementations of the proposed framework: (1) improve the tracking accuracy of RFtracker with local refinement and without regard to dynamic occlusion (referred to as RFtracker-A); (2) an integrated implementation with both local refinement and the occlusion detection module (referred to as RFtracker-B). Furthermore, the re-implementation is referred to as RFtracker*. Both RFtracker-A and RFtracker-B build on this re-implementation. In this experiment, we employ a dataset consisting of 4 synthetic and 2 real RGB-D image sequences with ground truth transformation. As in [17], four synthetic object (Kinect box, tide, orange juice, milk) sequences are chosen for evaluation in terms of tracking accuracy, i.e., errors in translation and rotation. We compare RFtracker*, RFtracker-A, and RFtracker-B with the original RFtracker. Here, median errors of translation (mm) and rotation (degrees) are given in Table 1. Note that mean errors of four sequences corresponding to RFtracker are provided by the authors [44].

From the results listed in Table 1, the tracking accuracy of the RFtracker* is comparable to the original version. After integrating the local refinement module, RFtracker-A achieves the best mean error in translation. Compared with RFtracker*, RFtracker-A has achieved better results on all indicators. Note that the tracking accuracy of RFtracker-B is slightly worse than that of RFtracker-A because of the non-occluded attribute of LineMod sequences. The CMC module filters out both the background pixels and parts of high-quality areas. Even so, mean errors of RFtracker-B are still better than RFtracker*.

### 3.3. Tracking Accuracy on a Real Dataset

**Figure 8:**
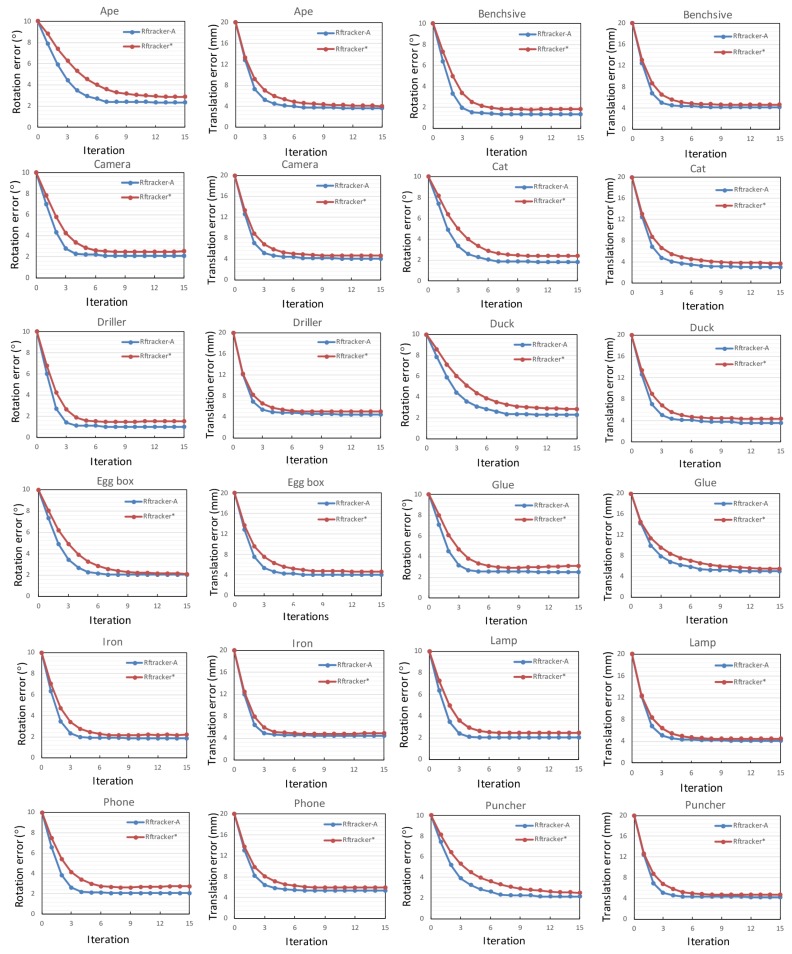
Comparison of tracking accuracy against the number of iterations. All the test objects are from the LineMod dataset [23].

For the synthetic dataset, the rendering engine is idealized without distortion and noise. Hence, we utilize the extensive ACCV (Asian Conference on Computer Vision) dataset [23] for further evaluation in terms of tracking accuracy in this experiment. This real dataset consists of over 18,000 real images with 15 different objects and the ground truth pose. Note that three of the LineMod objects (bowl, can, cup) are removed for lacking the mesh model. Furthermore, this dataset aims to evaluate the accuracy of object detection and pose estimation in heavily-cluttered scenes and does not have a specially-designed occluded scene to evaluate the robustness of algorithms. In this experiment, we only examine the lifting tracking accuracy of the local refinement module. Similar to [17], the convergence rate describing average errors in translation and rotation with the increasing of iterations is employed as the evaluation criteria. Corresponding plots of both RFtracker* and RFtracker-A on each LineMod sequence are shown in Figure 8. The ground truth pose of each frame is combined with a relative transformation of the translation range in $\left[-20\;\mathrm{mm},\;20\;\mathrm{mm}\right]$ and the rotation range in $\left[-10^{\circ },\;10^{\circ }\right]$ to mimic the previous transformation.

For the LineMod dataset, RFtracker-A achieves better results than the reimplementation on all sequences. Specific to each iteration, RFtracker-A can always converge to a lower error in both translation and rotation because of the local refinement module. Another nice bonus is that fewer iterations are required when RFtracker-A converges than RFtracker*. RFtracker-A takes only 6 or 7 iterations to converge.

### 3.4. Robustness against Occlusion

The framework’s robustness against occlusion is evaluated on a challenging benchmark dataset, the Occluded LineMod dataset. This dataset contains additional annotations for the original LineMod dataset [23], for which only ground truth poses for one object are given. Depending on the viewing direction, some objects are occluded by others. Thus, the Occluded LineMod dataset can provide us with heavily-occluded objects for which ground truth poses are available. In this experiment, we test all the sequence frames with all three methods, that is RFtracker*, RFtracker-A and RFtracker-B. To quantify the robustness against occlusion, the percentage of images where the object’s pose is estimated correctly is included. This protocol, which compares the average distance of all model vertices with the product of a scaling factor and the object’s diameter, was originally proposed by Hinterstoisser et al. [23]. The corresponding results are detailed in Table 2. Similar to the previous practice, the simulative input transformation is generated by combining the ground truth pose of each frame with an update transformation of the translation range in $\left[-20\;\mathrm{mm},\;20\;\mathrm{mm}\right]$ and the rotation range in $\left[-10^{\circ },\;10^{\circ }\right]$. Note that all the sampled update transformations are tested to ensure that the corresponding scaling factor is greater than 0.2.

The learning method with 3D object coordinates [21] is essentially a pose detection approach, which directly estimates the 6D pose of specific objects relative to the camera coordinate system. Therefore the data provided by this method are to determine roughly to which levels the proposed method belongs. As shown in Table 2, both RFtracker-A and RFtracker-B obtain more superior results than RFtracker*. Adding the local refinement module slightly improves the fraction of correct estimated frames by 0.7% on average. This indicates that the local refinement module does not have much inhibitory effect on noisy data mixed with the forest’s input. For *k* = 0.1, the lifting of the success rate is mainly from the improvement of tracking accuracy. Taking into account challenging scenes among the Occluded LineMod dataset, RFtracker-B significantly outperforms RFtracker* and RFtracker-A on this protocol by a large margin for both scaling factors. All of these benefit mainly from the occlusion handling module in the framework, which can efficiently filter out noisy data introduced by occlusions. The success rate of egg box being correctly estimated is extremely low because egg box is out of view and heavily occluded in numerous scenarios.

**Figure 9:**
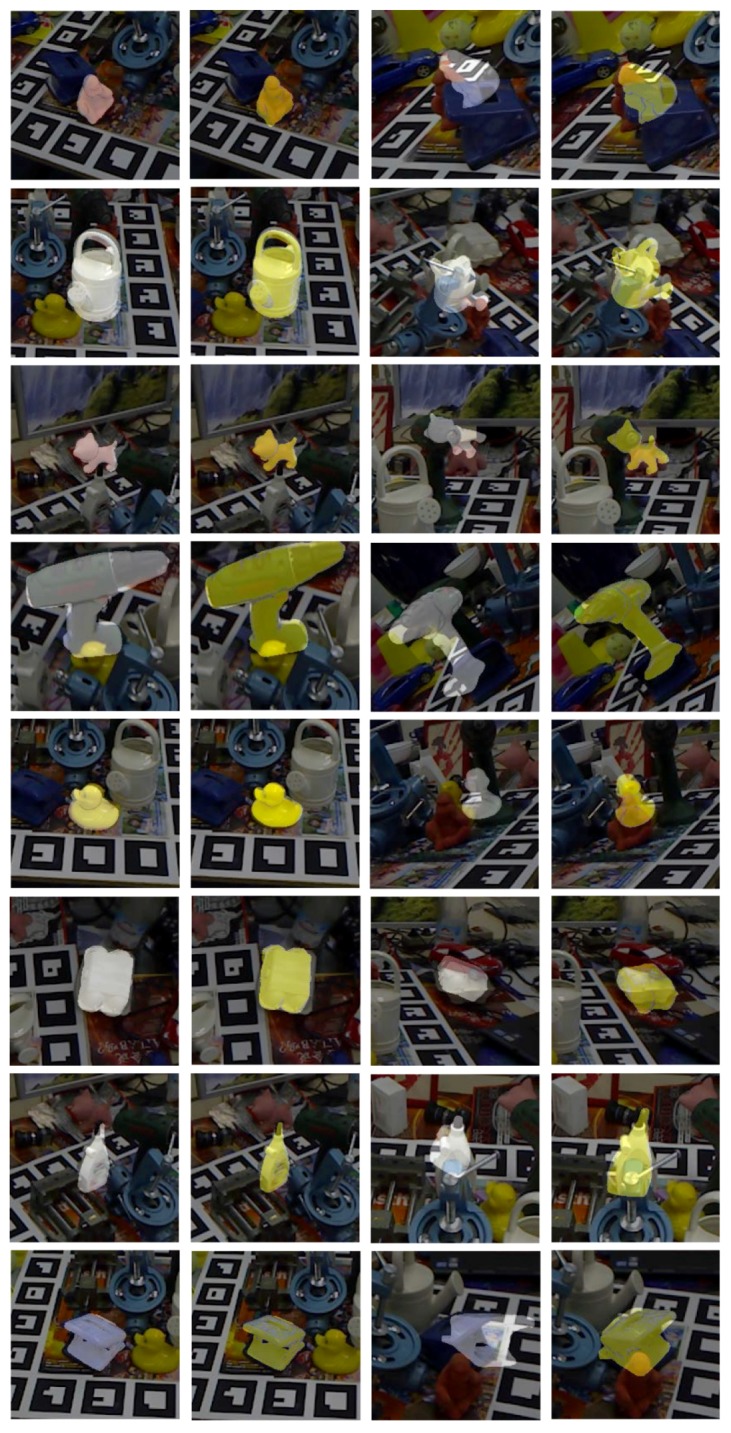
Example image crops showing the test results of RFtracker* (white) and RFtracker-B (yellow).

Some qualitative results on the Occluded LineMod dataset [23] are shown in Figure 9. The occlusion rate in the scenes corresponding to the two columns on the left side is low, while it is high in the two columns on the right side. From top to bottom, the eight tracked targets are ape, can, cat, driller, duck, egg box, glue and hole puncher, respectively. We show the projection mask of 3D rendering models on scene crops. The results of RFtracker* are shown in white, and our predictions (RFtracker-B) are shown in yellow. For the scenes for which the occlusion rate is low, both RFtracker* and RFtracker-B achieve visually satisfactory accuracy. For heavily-occluded scenes, RFtracker-B shows a stronger robustness against occlusion.

### 3.5. Computation Time

In the proposed framework, the random forest and local refinement are running on multi-core CPUs. The CMC module and occlusion detection are suitable for parallel computing, so GPU is employed to maximize the efficiency of our framework. Both the CMC module and the occlusion detection are executed once in each loop. By contrast, random forest and local refinement need to be iteratively executed multiple times until the interrupt condition is triggered. As shown in Table 3, the corresponding runtimes of different modules are listed.

Similar to [17], the runtime of our reimplementation (referring to RFtracker*) is less than 2 ms using the multi-core CPUs. The extra time overhead of RFtracker-A comes mainly from the local refinement module compared with RFtracker*. RFtracker-A runs at about 35 Hz on the LineMod sequences with a total of 7 iterations. When the CMC switch is closed, RFtracker-B achieves a runtime performance of more than 30 Hz. In cases when the CMC module is triggered by the update transformation between sequential frames, it goes down to about 25 Hz.

## 4. Conclusions

In the field of pose tracking, tracking accuracy and robustness to various interferences from the environment are still open problems to be further studied. Inspired by a strong baseline RFtracker, we have proposed an integrated framework with strong robustness to occlusion and improved precision. To address the disagreement between training and testing loss in the original RFtracker, a local refinement is introduced to take advantage of complementary information between adjacent trees. The evaluation on the LineMod dataset shows that the proposed refinement module can further improve the regression accuracy of the forest at the expense of less computational cost. The randomness of sample selection in the training data provides a built-in occlusion handling, which can deal with occlusion at primary levels. Thus, we propose an online rendering module with the CMC to label the low-quality region. The comparison with RFtracker shows that our method has stronger robustness to heavy occlusion. These improvements built on RFtracker allow our approach to maintain considerable accuracy under partial occlusions.

In future work, we will consider exploring a deep learning model to predict stable parts of a tracked object directly instead of the proposed occlusion handling module. Online rendering damages the scalable attribute of RFtracker, which is also an issue to be solved.

## Figures and Tables

**Table 1:** Comparison of tracking accuracy on the synthetic dataset [22]. The errors are denoted in translation as mm and rotation in degrees. Optimal results for each term are in bold.

Method	Kinect Box		Tide		Orange Juice		Milk		Mean	
	tran.	rot.	tran.	rot.	tran.	rot.	tran.	rot.	tran.	rot.
RFtracker [17]	1.70	**0.3**	**1.17**	0.44	**1.29**	**0.35**	1.27	**0.41**	1.36	**0.37**
RFtracker*	1.67	0.53	1.29	0.50	1.49	0.42	1.19	0.69	1.41	0.54
RFtracker-A	**1.40**	0.45	1.19	**0.41**	1.35	0.40	**1.08**	0.54	**1.26**	0.45
RFtracker-B	1.72	0.48	1.21	0.44	1.40	0.42	1.27	0.51	1.40	0.46

**Table 2:** Comparison of the Occluded LineMod dataset under two different scaling factors. We provide the percentage of frames for which the estimated average distance is smaller than the product of the scaling factor and the object’s diameter. Optimal results for different scaling factors are in bold. Avg. denotes the percentage here. RF*, RF-A and RF-B represent RFtracker*, RFtracker-A and RFtracker-B, respectively. Depth C. (depth component). denotes the method [21] with depth energy only.

Object	Depth C. [21]	RF*	RF-A	RF-B	Depth C. [21]	RF*	RF-A	RF-B
	k = 0.1				k = 0.2			
Ape	51.9	65.1	66.1	**68.3**	-	66.6	67.2	**70.0**
Can	**98.8**	74.9	75.9	78.0	-	77.6	79.2	**81.1**
Cat	27.7	37.2	37.7	**38.4**	-	38.3	39.0	**41.4**
Driller	**71.8**	59.6	60.3	62.7	-	61.2	62.5	**65.3**
Duck	57.8	62.7	63.1	**63.9**	-	63.3	64.3	**66.0**
Egg box	2.4	34.3	34.9	**35.7**	-	35.3	36.1	**38.3**
Glue	33.3	43.5	44.2	**45.6**	-	45.1	46.0	**47.8**
Hole puncher	71.5	73.5	74.0	**75.5**	-	74.9	75.5	**76.2**
Avg.	51.9	56.3	57.0	**58.5**	-	57.7	58.7	**60.8**

**Table 3:** Runtime analysis of the proposed framework.

	Processing step	Time
per frame	CMC module	7 ms
	Occlusion detection	4 ms
per iteration	Random forest + local refinement	4 ms
	RFtracker-A	4×7=28 ms
	RFtracker-B with CMC off	4×7+4=32 ms
	RFtracker-B with CMC on	4×7+4+7=39 ms
